# Supplementary material for: Controlled dissolution of a single ion from a salt interface
Source: Nat Commun. 2024 Mar 16;15:2401. doi: 10.1038/s41467-024-46704-y (PMC10944500; doi:10.1038/s41467-024-46704-y)
Supplement: Supplementary file 1 — Supplementary Information [file 41467_2024_46704_MOESM1_ESM.pdf]

Supplementary Information for:

Controlled dissolution of a single ion from a salt interface

Huijun Han[1], Yunjae Park[2], Yohan Kim[1], Feng Ding[1,3]*, Hyung-Joon Shin[1,2]*

[1]Department of Materials Science and Engineering, Ulsan National Institute of Science and Technology (UNIST), Ulsan 44919, Republic of Korea.

[2]Center for Multidimensional Carbon Materials, Institute for Basic Science (IBS), Ulsan 44919, Republic of Korea.

[3]Shenzhen Institute of Advanced Technology, Chinese Academy of Science, Shenzhen 518055, China.

*To whom correspondence should be addressed.

Email: f.ding@siat.ac.cn (F.D.)

Email: shinhj@unist.ac.kr (H.-J.S.)

Table of contents

Supplementary Notes 1 to 3

Supplementary Figs. 1 to 8

Supplementary References

Supplementary Note 1: Fuzzy features of H_2O at the step

The water molecules at the step showed a fuzzy feature in the STM topography despite the higher adsorption energy of the molecule at the step than on the terrace. The DFT calculations show that the water molecule is not located exactly at the top of Na^+ site but slightly shifted from a Na^+ site. There are two local energy minimums at the step (state 3 and 19 in Supplementary Fig. 5). The energy barrier between the minimums is about 43.6 meV. The fuzzy feature at the step is attributed to continuous switching between two configurations (state 3 ↔ state 19).

Supplementary Note 2: Direct extraction of Cl^- ions

There have been some reports regarding direct extraction of Cl^- ions using STM tip[1-5]. In this study, we tried to minimize other factors that could affect the dissolution process, as our major goal was to extract single ions by manipulating water molecules. In order to investigate the role of a water molecule in the selective dissolution, we tried transferring Cl^- ions from NaCl surface to the STM tip[1-5], which was impossible on Ag(100) even with severe tunnelling conditions (V_s = 5 mV, I_t = 3.0 to 5.0 nA). From the NaCl films on 'Au(111)' or 'Cu(111)', however, we could extract ions by applying a voltage pulse[1-5]. It means that a water molecule plays an important role in the dissolution reaction of NaCl films in our results.

Supplementary Note 3: Identifying the type of vacancy at the step

Na^+ ions of NaCl surface are invisible in STM mode due to its low density of state. In recent works by Jiang's group[2,6], a single vacancy is observable as a depression at a Na^+ site. As we did not see any depressions or vacancies at Na^+ sites, we confirmed that there is no dissolved Na^+ ion during the manipulation. If a NaCl pair is removed from the step, the shape of vacancy should be asymmetric; however, our result showed that the vacancy has a symmetric feature at the Cl^- site (Fig. 5d). We simulated the STM images of the possible vacancies at the step (Na^+, Cl^- and NaCl pair) using DFT calculations (Supplementary Fig. 8). The symmetric feature of the vacancy in Fig. 5d agrees well with the symmetric shape of a single Cl^- vacancy in the simulated image, which supports the preferential dissolution by a single water molecule.

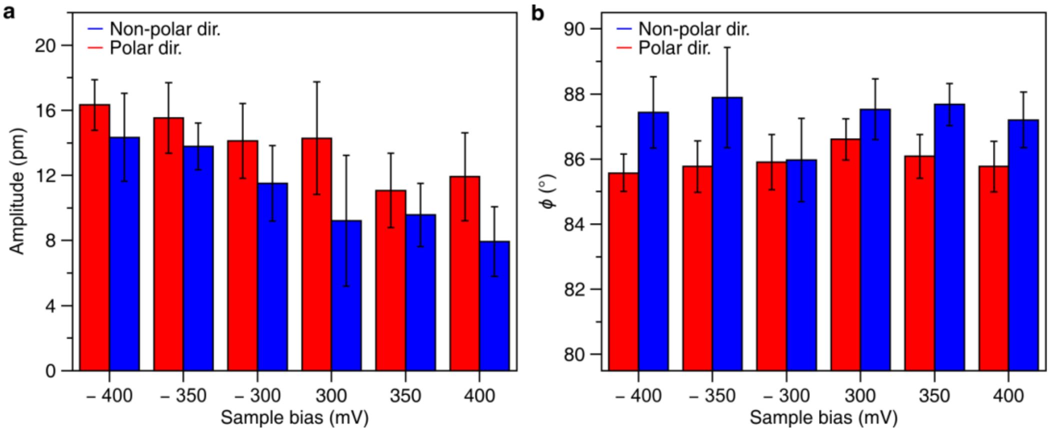

Supplementary Fig. 1 | Quantitative analysis of the lateral manipulation. a, Averaged amplitudes of tip height profiles. **b**, Averaged angles from tip height profiles. These data were derived from the manipulation profiles. Tunnelling current was 240 and 80 pA at positive and negative bias voltages, respectively. Each data was averaged over at least eight trials. The amplitude along the non-polar direction is lowered than that along the polar direction, while the angle along the non-polar direction is higher than that along the polar direction. The error bars indicate the standard deviation.

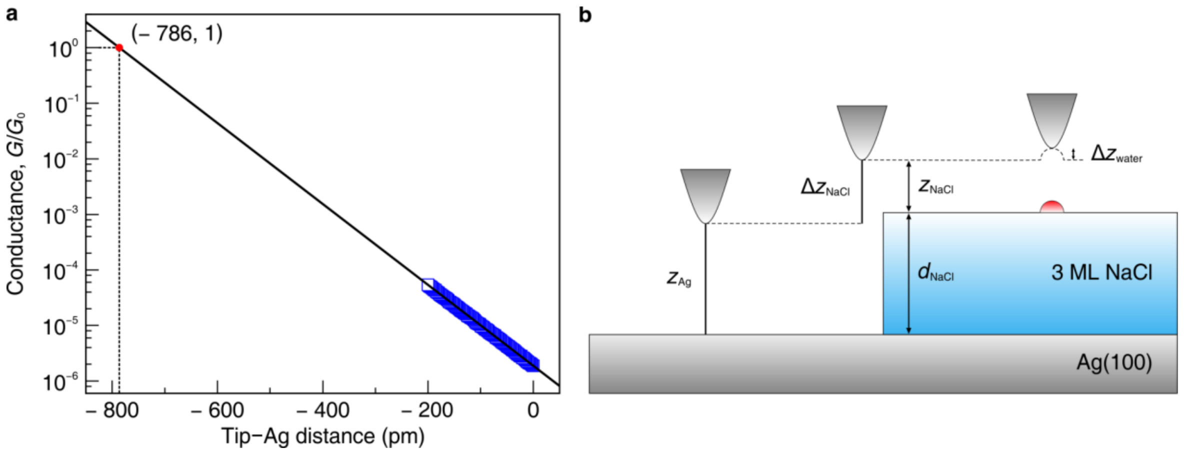

Supplementary Fig. 2 | Determination of the tip-Ag surface distance. a, Conductance versus tip height derived from *I-z* spectroscopy on the Ag surface (V_s = 350 mV, I_t = 50 pA). G_0 is the quantization conductance, $2e^2/h$ with the electron charge e and Planck constant h. The black line is an extrapolated line from the least-squares fitting of the data (blue squares). The line extends to the contact point where the tip approached the surface at 786 pm. **b**, Height scheme of a single water molecule on the 3 ML NaCl film. z_{Ag} and z_{NaCl} represent the tip height on the Ag substrate and the NaCl film, respectively. Δz_{NaCl} and Δz_{water} are the apparent heights of the film and the molecule, respectively. d_{NaCl} is the physical height of the film.

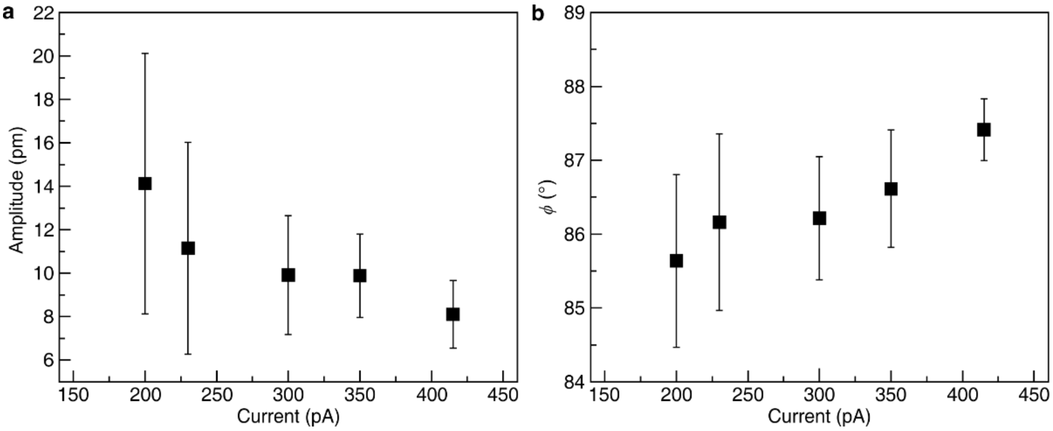

Supplementary Fig. 3 | Effect of tip with various currents on lateral manipulation. a, Averaged amplitudes of tip height profiles measured from polar direction with various current at the bias voltage of 350 mV. **b**, Averaged angles obtained from tip height profiles. Each point was averaged over at least fifteen trials. The error bars indicate the standard deviation.

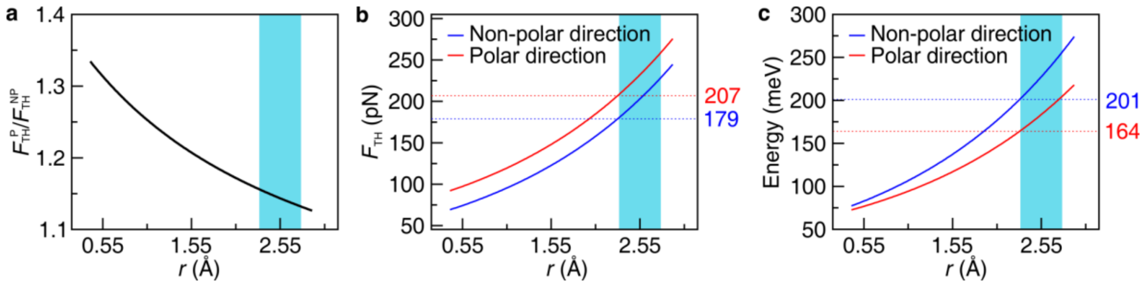

Supplementary Fig. 4 | Effect of tip on lateral manipulation. a, $F_{\mathrm{TH}}^{\mathrm{P}}/F_{\mathrm{TH}}^{\mathrm{NP}}$ as a function of *r*. **b**, Forces along the polar and non-polar directions. **c**, Energy barriers of the polar and non-polar directions. Dashed lines in **b** and **c** are threshold frictional forces and energy barriers without the presence of tip. Blue areas show the range of tip-molecule distance where the present lateral manipulations were performed.

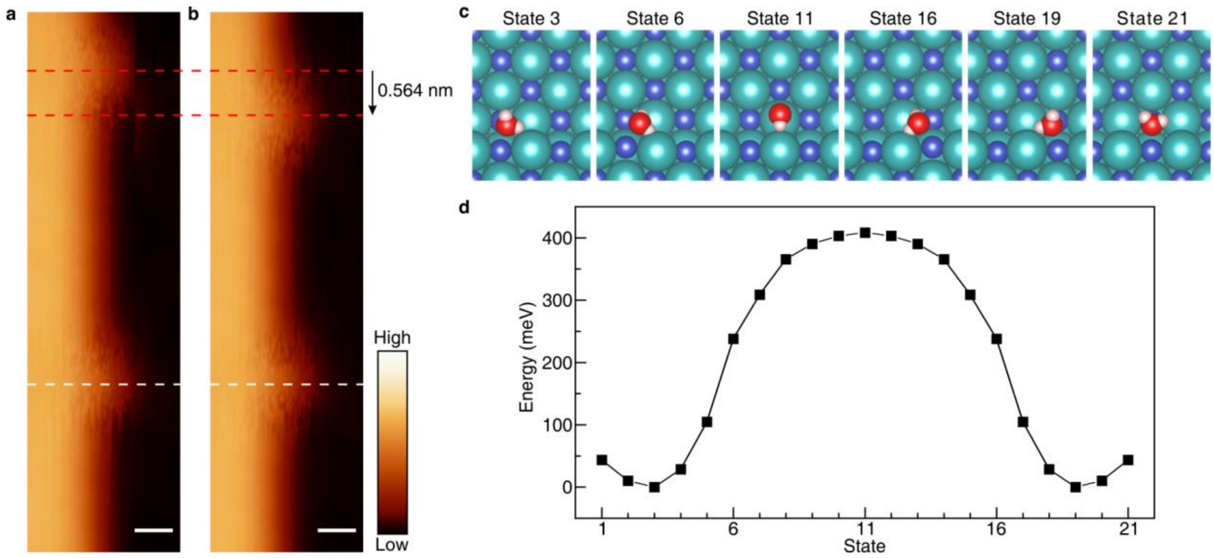

Supplementary Fig. 5 | Lateral manipulation of a single water molecule at a step. a, Two water molecules at the step edge before manipulation (V_s = −350 mV and I_t = 50 pA). **b**, The water molecule moved along the arrow. Scale bars in **a** and **b** represent 0.5 nm. The displacement was 0.564 nm. **c**, Representative optimized molecular configurations along the non-polar step calculated by DFT. **d**, Energy profile of the water molecule along the step edge.

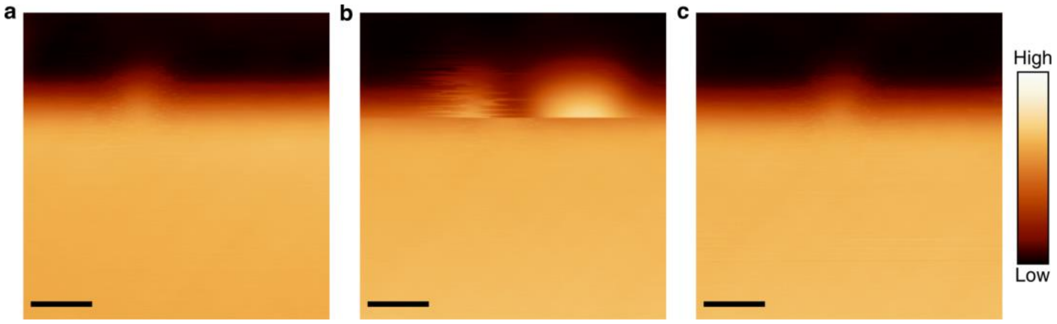

Supplementary Fig. 6 | Recovery of Cl^- vacancy with the extracted anion. a, A single water molecule at the step before manipulation. **b**, After manipulation, the anion was extracted and adsorbed near the vacancy with the water molecule. **c**, Manipulation of the anion into the vacancy. Scale bars in **a**–**c** represent 1 nm. All images were taken under V_s = –350 mV and I_t = 50 pA. Sequential images show that the depression was the Cl^- vacancy, not the dissociated species of a water molecule.

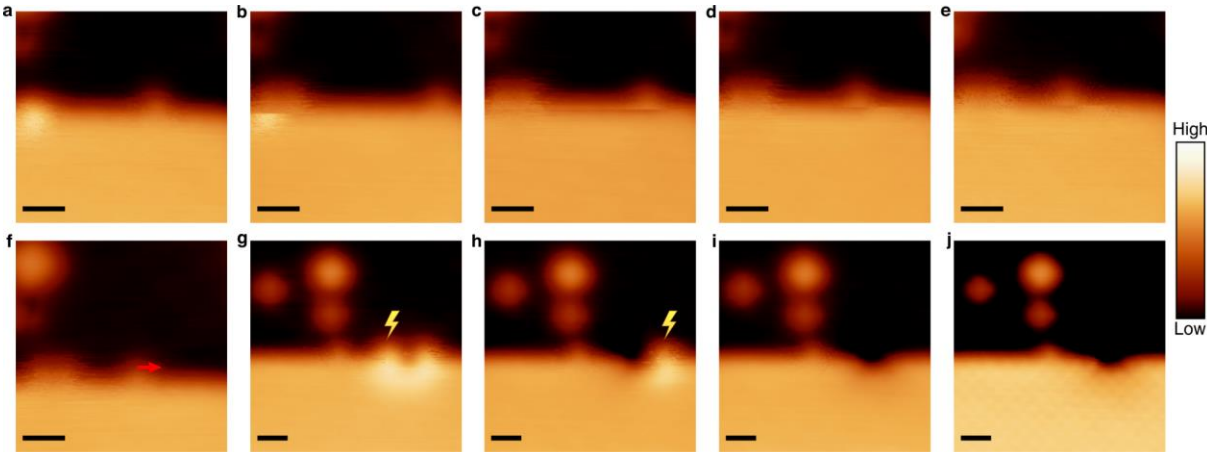

Supplementary Fig. 7 | Consecutive manipulations for selective dissolution in Fig. 5d. a, STM image of the 2 ML/3 ML interface with water molecules (V_s = −300 mV and I_t = 50 pA). **b**, Adsorption of two water molecules from a water dimer at the step (V_s = −350 mV and I_t = 50 pA). **c**–**e**, Consecutive manipulations of a single water molecule at the step (V_s = −350 mV and I_t = 50 pA). **f**, Manipulation of the molecule along the red arrow (V_s = −350 mV and I_t = 50 pA). **g**, STM image after manipulation (V_s = 400 mV and I_t = 50 pA). Two new fuzzy features appeared in a symmetric configuration at the location of molecular manipulation. As the left water molecule was not dissociated during the manipulation, it was desorbed by applying a 600−meV tunnelling electron. **h**, Successive applying a bias voltage to the right water molecule (V_s = 400 mV and I_t = 50 pA). **i**, STM image of a single Cl^- vacancy (V_s = 400 mV and I_t = 50 pA). The extracted Cl^- disappeared after the manipulation. **j**, High-resolution STM image of **i** (V_s = 400 mV, I_t = 500 pA). Scale bars in **a**–**j** represent 1 nm.

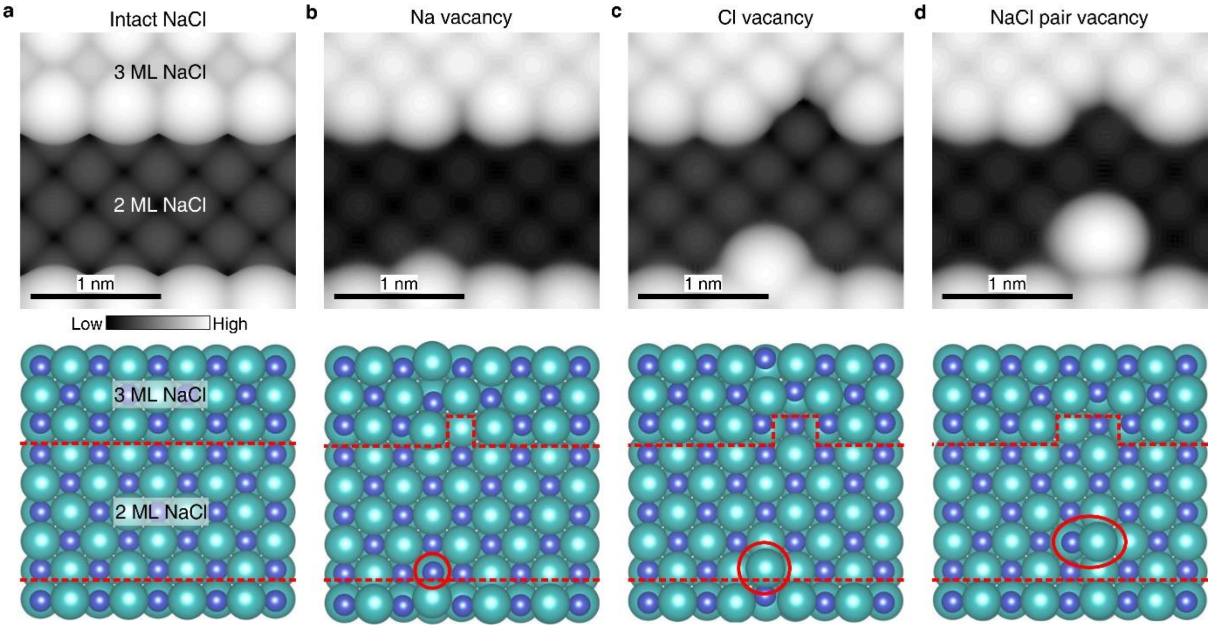

Supplementary Fig. 8 | Simulated STM images of perfect NaCl step edge and the step edge with different type of defects. **a**, Simulated STM image of intact NaCl surface with two parallel step edges (top) and the corresponding atomic model (bottom). **b**, Simulated STM image of a Na vacancy on the step edge (top) and the corresponding atomic model (bottom). **c**, Simulated STM image of a Cl vacancy on the step edge (top) and the corresponding atomic model (bottom). **d**, Simulated STM image of a NaCl pair vacancy on the step edge (top) and the corresponding atomic model (bottom). In **c**, the symmetric structure of a single Cl vacancy matches the experimental STM image in Fig. 5d.

Supplementary references

1. Repp, J., Meyer, G., Paavilainen, S., Olsson, F. E. & Persson, M. Scanning tunneling spectroscopy of Cl vacancies in NaCl films: Strong electron-phonon coupling in double-barrier tunneling junctions. *Phys. Rev. Lett.* **95**, 225503 (2005).
2. Peng, J. *et al*. The effect of hydration number on the interfacial transport of sodium ions. *Nature* **557**, 701–705 (2018).
3. Schuler, B. *et al*. Effect of electron-phonon interaction on the formation of one-dimensional electronic states in coupled Cl vacancies. *Phys. Rev. B* **91**, 235443 (2015).
4. Li, Z. *et al*. Lateral manipulation of atomic vacancies in ultrathin insulating films. *ACS Nano* **9**, 5318– 5325 (2015).
5. Meng, X. *et al*. Direct visualization of concerted proton tunnelling in a water nanocluster. *Nat. Phys.* **11**, 235-239 (2015)
6. Peng, J., Guo, J., Ma, R., Meng, X. & Jiang, Y. Atomic-scale imaging of the dissolution of NaCl islands by water at low temperature. *J. Phys.: Condens. Matter* **29**, 104001 (2017).
